# Plant resting site preferences and parity rates among the vectors of Rift Valley Fever in northeastern Kenya

**DOI:** 10.1186/s13071-016-1601-7

**Published:** 2016-05-31

**Authors:** Samwel O. Arum, Christopher W. Weldon, Benedict Orindi, Caroline Tigoi, Francis Musili, Tobias Landmann, David P. Tchouassi, Hippolyte D. Affognon, Rosemary Sang

**Affiliations:** International Centre of Insect Physiology and Ecology, Nairobi, P. O. Box 30772-00100, Nairobi, Kenya; Department of Zoology and Entomology, University of Pretoria, Private Bag X20, Hatfield, 0083 South Africa; International Crops Research Institute for the Semi-Arid Tropics (ICRISAT), BP 320, Bamako, Mali

**Keywords:** Parity, Survival, Resting preference, Vegetation, Rift Valley fever vectors

## Abstract

**Background:**

Mosquito lifespan can influence the circulation of disease causing pathogens because it affects the time available for infection and transmission. The life-cycle of mosquitoes is determined by intrinsic and environmental factors, which can include the availability of hosts and suitable resting environments that shelter mosquitoes from extreme temperature and desiccating conditions. This study determined the parity rates (an indirect measure of survival) and plant resting preference of vectors of Rift Valley fever (RVF) in northeastern Kenya.

**Methods:**

Resting mosquitoes were trapped during the rainy and the dry season using a Prokopack aspirator from vegetation, whereas general adult populations were trapped using CDC light traps. At each site, sampling was conducted within a 1 km^2^ area, subdivided into 500 × 500 m quadrants and four 250 × 250 m sub-quadrants from which two were randomly selected as sampling units. In each sampling unit, plants were randomly selected for aspiration of mosquitoes. Only *Aedes mcintoshi* and *Ae. ochraceus* were dissected to determine parity rates while all mosquito species were used to assess plant resting preference.

**Results:**

Overall, 1124 (79 %, 95 % CI = 76.8–81.1 %) mosquitoes were parous. There was no significant difference in the number of parous *Ae. mcintoshi* and *Ae. ochraceus*. Parity was higher in the rainy season than in the dry season. Daily survival rate was estimated to be 0.93 and 0.92 among *Ae. ochraceus* and *Ae. mcintoshi*, respectively. *Duosperma kilimandscharicum* was the most preferred plant species with the highest average capture of primary (3.64) and secondary (5.83) vectors per plant, while *Gisekia africana* was least preferred.

**Conclusion:**

Survival rate of each of the two primary vectors of RVF reported in this study may provide an indication that these mosquitoes can potentially play important roles in the circulation of diseases in northern Kenya. Resting preference of the mosquitoes in vegetation may influence their physiology and enhance longevity. Thus, areas with such vegetation may be associated with an increased risk of transmission of arboviruses to livestock and humans.

## Background

Mosquito life-cycles are important in determining their vectorial capacity in transmission of vector-borne diseases such as Rift Valley fever (RVF). Transmission and circulation of pathogens by mosquitoes is influenced by a range of biological factors including mosquito distribution, host preferences, resting behaviour and availability of suitable environmental conditions [1]. Prevailing environmental conditions, in particular, are key to the circulation of vector-borne diseases by insects because they dramatically influence the life history traits of vectors such as development rate and population growth rate.

The lifespan of mosquito vectors is highly variable, depending on species and environmental conditions [2, 3]. The average lifespan of an adult mosquito species can vary from 13 to 20 days. In *Anopheles* species found in the tropics, this duration can vary between 10–14 days but may occasionally reach 21 days [4]. Some mosquitoes are also known to live longer by entering dormancy to avoid unsuitable weather periods [5]. Other mosquitoes hibernate during the winter as adults, so their lifespan as full-grown mosquitoes lasts for months [6]. It is also known that temperature and its interaction with age and sex is an important variable that determines the lifespan of mosquitoes [7].

It is often not practical to directly measure the lifespan of wild mosquitoes. However, some studies have conducted indirect estimates of daily survivorship for anopheline mosquitoes as a means of estimating their longevity [8]. Morphological methods can be performed using laboratory technology, such as fine dissection tools and a light microscope. However, the dissection techniques can be labour intensive when handling large numbers of samples. The age grading techniques currently used include Detinova ovarian tracheation (parity), Polovodova ovariole dilatation, and daily growth line methods. Parity can also be used to estimate the daily survival rate of mosquitoes following the Davidson method using the proportion of parous mosquitoes [9]. The most commonly applied morphological classification technique for mosquitoes has been the ovary tracheation method of Detinova [10]. This technique proved successful in grading of mosquitoes as parous and nulliparous for comparison of parity rates in mosquitoes [11].

Outdoor and indoor resting habits are common among mosquito vectors. However, it is unclear whether mosquitoes that rest outdoors prefer certain vegetation or resting sites. The knowledge of the resting sites of different mosquito species is important for targeted vector control, and may optimise sampling of fed mosquitoes for studies on their host preferences [12–14]. Studies have documented variability in resting preference among mosquito species with some known disease vectors showing variable resting tendencies including endophilic and exophilic resting behaviour [15, 16]. However, most studies conducted on parity and resting of mosquitoes have focused on vectors of malaria as opposed to vectors of other diseases such as RVF. Vectors of RVF such as *Aedes* (*Neomelanconion*) *mcintoshi* Huang and *Aedes* (*Aedimorphus*) *ochraceus* (Theobald) are widely distributed in diverse ecological zones in northern Kenya and have played an important role in transmission of the arbovirus in this region [17]. When an infected blood meal is successfully obtained by these vectors, an incubation period is required before they can successfully transmit the disease [18, 19]. However, the presence and abundance of these mosquitoes at any given time may also be influenced by a number of factors such as climatic variables and parasite burden [20–23].

Northeastern Kenya is primarily dominated by large grazing areas occupied by nomadic pastoralists. The landscape supports a wide array of diverse plant species that could provide ideal resting habitats for mosquitoes. Identifying preferred resting habitats could guide novel adult vector control efforts during outbreaks as a means of breaking transmission cycles. It may identify plants attractive or repellent to mosquitoes [24, 25]. Information regarding resting preference of RVF vectors in natural environments remains unknown, frustrating efforts to break transmission cycles during outbreaks using chemical control of adult populations. Even though sampling for outdoor resting mosquitoes can be complex [26], studies on outdoor resting behaviour of RVF vectors remain an important component in managing RVF outbreaks. The results of such studies will assist in determining potential habitats used by mosquitoes for resting, which may then be targeted for vector control. This study examined parity rates among the key primary vectors of RVF, *Ae. mcintoshi* and *Ae. ochraceus*, and the resting preference of RVF vectors among different plant species. The survival rate of wild-caught *Ae. mcintoshi* and *Ae. ochraceus* was estimated by determining the parity of females. These data will provide insights into mosquito behaviour under field conditions that can be used for focused and site specific vector control to minimize the transmission of RVF and other diseases borne by these mosquito species.

## Methods

### Study area

**Fig. 1:**
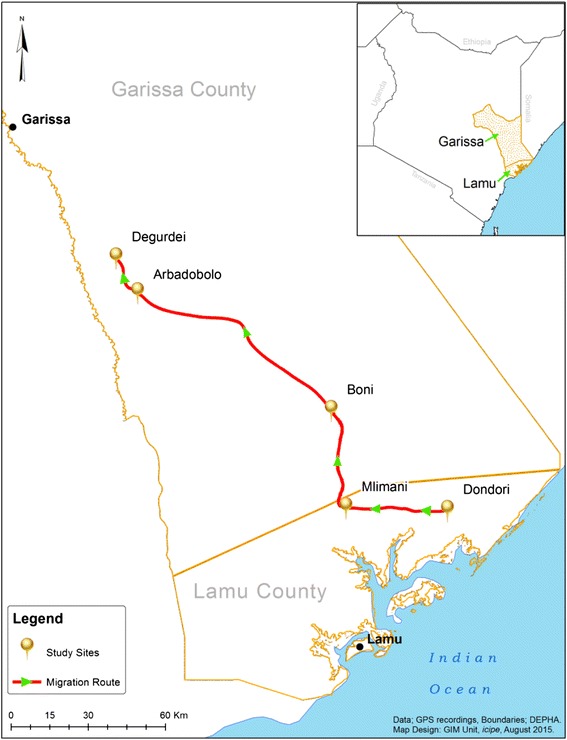
Map of Kenya showing the study sites along a livestock movement route

Mosquitoes were trapped from five sampling sites: Degurdei, Arbadobolo, Boni, Mlimani, and Dondori in Garissa (00°39'S, 40°05'E) and Lamu (02°16'S, 40°12'E) counties in the north-eastern region of Kenya (Fig. 1). These sites were located within three distinct ecological zones: semi-arid, dry humid forest and humid to dry sub-humid. The region is inhabited by the ethnic Somali community whose main source of livelihood is nomadic pastoralism. Annual livestock migration in the region occurs due to scarcity of rainfall. This region is known for the abundance of mosquitoes during the rainy seasons and risk of exposure to mosquito-borne diseases*.* Climatic conditions range from extreme dry weather to extreme flooding conditions during the rainy seasons. The mean annual rainfall varies between 200 and 500 mm. Rainfall pattern is bimodal, with two rainy seasons from April to May, and between October and November with occasional variation. Generally, the region is hot and dry with average daily temperatures ranging from 20 to 38 °C. There is usually rapid growth of vegetation at most sites during the rainy season, which may form suitable resting habitats for mosquitoes.

### Sampling mosquitoes from vegetation

Mosquitoes were captured from vegetation to determine potentially preferred resting sites. There were no built structures at any of the sampling sites that could act as alternative mosquito resting sites. At each of the five sampling sites, a 1 km^2^ sampling area was identified and subdivided into four quadrants (A,B,C,D) each measuring 500 × 500 m. Each of the four quadrants was subdivided into four sub-quadrants (A1-4, B1-4, C1-4, D1-4) that were 250 × 250 m each totaling to 16 sub-quadrants. Two of the sub-quadrants were randomly selected as sampling units for mosquito collection using two CDC light traps (LTs) and direct aspiration from vegetation. Mosquitoes were trapped during the rainy seasons (April-June) and dry seasons (August-October) in 2013 and 2014. In total, eight light traps and eight sub-quadrants were sampled at each site, on each sampling event. The LTs were set at 18:00 h and retrieved at 06:00 h the next morning taking into account the activity of these vectors. A Prokopack aspirator (John W. Hock Company) [27], was used to separately trap resting mosquitoes from four randomly selected plants in each of the 8 randomly selected sub-quadrants in each site. Only plants that yielded mosquitoes were considered for analysis of mosquito resting preference. Resting mosquitoes were aspirated between 14:00 h and 16:00 h. Aspiration from all selected plant species in each quadrant was conducted for 20 min. Mosquitoes were identified morphologically using the keys of Edwards [28] and the plants from which mosquitoes were captured were collected and labelled for identification with the help of a botanist.

### Determination of parity of the key vectors of RVF

Trapped mosquitoes were used to determine parity rates among the primary vectors of RVF. *Ae. mcintoshi* and *Ae. ochraceus* were the only vector species dissected to determine parity rates. A dissected mosquito was classified as parous when it showed evidence of previous blood feeding and egg production, or nulliparous for a mosquito without evidence of previous blood feeding and egg production. The mosquitoes were individually dissected with the aid of a stereomicroscope with further observation of the ovary using a compound microscope (Leica DMRB). Anaesthetised adult females were gently placed on a clean microscope slide and dissected into a drop of phosphate-buffered saline (PBS) [10]. During dissection, the thorax was gently held by forceps and placed ventral side up with its abdomen in the phosphate buffered saline. A fine tip needle was used to gently remove the 7th and 8th abdominal segments by grasping and gently pulling them away. Ovaries appeared as a pair of white oval objects attached to the removed segments, which were isolated, transferred to a new slide and allowed to air-dry. The dry specimen was viewed under a compound microscope to determine the overall number of parous and nulliparous individuals among all dissected *Ae. mcintoshi* and *Ae. ochraceous*.

### Data analysis

To investigate the plant species preferred as resting sites by mosquitoes, we used a negative binomial model for all mosquito species combined and separately for each mosquito species, with plant species and season as covariates in the model. Risk ratios (RR) were computed for each plant species in comparison to *Duosperma kilimandscharicum* Clarke, which was the most preferred plants species. All analyses were performed using R v3.2.0 [29]. There was no association between the collection method and species caught (Chi-square test; *χ*^2^ = 1.11, *df* = 1, *P* = 0.291), so we analysed data from the two collection methods (i.e. LT and aspiration) together. Data on the number of parous and nulliparous mosquitoes were compared using a quasibinomial model with vector species (where *Ae. mcintoshi* = 0, *Ae. ochraceus* = 1), season (rainy season = 0, dry season = 1) and collection method (aspiration = 0, LT = 1) as variables in the model. Odds ratios (OR) were computed to quantify the effect of each variable. To compare survival rate between the mosquitoes, daily survival rate was estimated based on the proportion of individuals that were parous using the formula *p*^*n*^ = M where (*p)* is the survival rate per day, (M) is the proportion of the population which is parous and (*n*) is the number of days between emergence of adult and first oviposition as described by Davidson [9]. This method was adapted based on the following assumptions: (i) It is assumed that the population is static over the sampling period, i.e. that births equal deaths and immigration equals emigration; and (ii) It is assumed that the instantaneous mortality rate of the population is constant at all ages. For both species our study also assumed that *n* value = 3, given that the information on the species in question have not been documented in literature and could not be determined experimentally in the laboratory.

## Results

### Mosquito diversity and abundance

Seven mosquito species belonging to two main genera (*Aedes* and *Culex*) were trapped during this study. Overall 1653 mosquitoes were aspirated from 192 plants that yielded mosquitoes (mean = 8.61, SD = 5.51 per plant). These plants comprised 12 plant species classified under 11 plant families. As noted earlier, the primary vectors of RVF*,* were also caught in large numbers from plants. In total, 1422 primary vectors of RVF, *Ae. mcintoshi* (46 %) and *Ae. ochraceus* (54 %) were captured by LT (*n* = 937) and aspiration (*n* = 485) methods. In addition, the secondary vectors *Ae.* (*Mucidus*) *sudanensis* (Theobald) (*n* = 149), *Cx.* (*Culex*) *pipiens* L (*n* = 149), *Cx.* (*Oculeomyia*) *poicilipes* (Theobald) (*n* = 131) and *Cx.* (*Culex*) *univittatus* (Theobald) (*n* = 202), *Cx. ethiopicus* (*n* = 51) and *Ae.* (*Aedimorphus*) *tricholabis* Edwards (*n* = 250) were also caught.

### Resting preference among RVF vectors

The mosquitoes aspirated from plants comprised both primary and secondary vectors of RVF. The different plant species that were sampled were resting sites to different proportions of the total number of mosquitoes that were caught: *Duosperma kilimandscharicum* Clarke (Acanthaceae) 39.1 %, C*ommelina forskali* Vahl (Comelinacea) 13.0 %, *Salsola kali* L. (Amaranthaceae) 10.9 %, *Salvadora persica* Kharija (Salvadoraceae) 10.9 %, *Cadaba ruspolii* Gilg (Capparaceae) 8.9 %, *Mollugo nodicaulis* Lam (Molluginaceae) 4.7 %, *Cyperus giolii* Clarke (Cyperaceae) 4.7 %, *Grewia tenax* Vahl (Malvaceae) 4.2 %, *Gisekia africana* Lour (Gisekiacea) 2.1 %, *Polygola erioptera* (Polygalaceae) 0.5 %, *Mollugo cerviana* (L.) (Molluginaceae) 0.5 %, and *Momordica rostrata* (Zimm) (Cucurbitacea) 0.5 %.

**Fig. 2:**
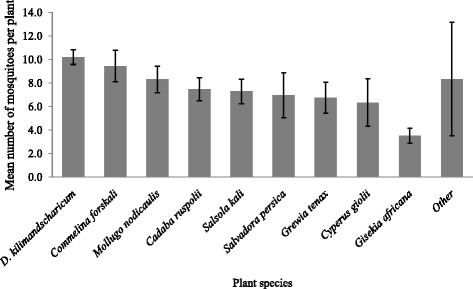
Mean number of mosquitoes captured per single plant species

**Fig. 3:**
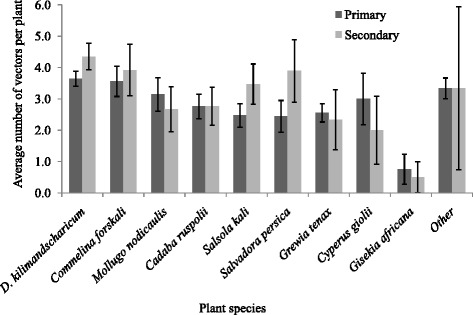
Mean number of primary and secondary RVF vectors captured per plant

We used the mean mosquito catches per individual plant species to determine the general plant resting preference for each mosquito species. Mosquitoes were most often captured from *D. kilimandscharicum* (*n* = 972), while the lowest number was captured from *G. africana* (*n* = 27)*.* Although the average mosquito captured per plant was higher for *D. kilimandscharicum* than any other plant species, the difference was minimal (Fig. 2). The average catches of primary and secondary RVF vectors also showed differences in plant resting preference between the two key RVF vector groups. Overall, *D. kilimandscharicum* was the most preferred by both vector groups, while the lowest average catches were from *G. africana* (Fig. 3). Secondary vectors were more abundant than primary vectors for most plant species, except for *M. cerviana* and *M. rostrata* from which none of the secondary vectors were captured.

For all mosquito species combined, the first column of Tables 1 and 2 shows that, compared to *D. kilimandscharicum* and after controlling for season, *S. kali, S. persica, C. ruspolii* and *G. africana* were significantly less preferred whereas *C. forskali, M. nodicaulis* and *G. tenax* were equally preferred to *D. kilimandscharicum.* There were, on average, significantly more mosquitoes caught from vegetation during the dry season than in the rainy season (RR = 1.27, 95 % CI = 1.05–1.53, *P* = 0.012). However, the difference in captures per plant between the seasons was not significant among individual vector species captured except for *Ae. sudanensis* in which significantly higher numbers were caught from vegetation during the dry season (RR = 2.17, 95 % CI = 0.67–1.64, *P* < 0.001) (Tables 1 and 2).

**Table 1:** Plant resting preference by *Aedes* spp.: negative binomial regression model results including a summary for *Aedes* spp. and *Culex* spp. combined

Variables	Mosquito species									
	*All species combined*		*Ae. mcintoshi*		*Ae. ochraceus*		*Ae. tricholabis*		*Ae. sudanensis*	
	RR (95 % CI)	*P*	RR (95 % CI)	*P*	RR (95 % CI)	*P*	RR (95 % CI)	*P*	RR (95 % CI)	*P*
Plant species										
*D. kilimandscharicum*	1		1		1		1		1	
*Commelina forskali*	0.93 (0.71–1.22)	0.605	0.88 (0.59-1.29)	0.521	1.15 (0.76–1.72)	0.496	0.83 (0.44–1.6)	0.577	0.82 (0.44–1.60)	0.551
*Salsola kali*	0.69 (0.51–0.93)	0.014	0.56 (0.34–0.88)	0.015	1.29 (0.84–1.95)	0.238	0.48 (0.22–1.02)	0.054	0.35 (0.22–1.02)	0.021
*Salvadora persica*	0.66 (0.48–0.90)	0.008	0.71 (0.45–1.11)	0.140	0.80 (0.48–1.30)	0.379	0.60 (0.29–1.26)	0.173	0.95 (0.29–1.26)	0.886
*Cadaba ruspolii*	0.71 (0.51–0.99)	0.040	0.66 (0.40–1.06)	0.095	0.67 (0.37–1.15)	0.165	0.70 (0.33–1.53)	0.366	1.12 (0.33–1.53)	0.752
*Mollugo nodicaulis*	0.79 (0.53–1.19)	0.252	0.68 (0.36–1.24)	0.224	0.61 (0.27–1.21)	0.186	0.38 (0.12–1.14)	0.084	1.22 (0.12–1.14)	0.641
*Cyperus giolii*	0.64 (0.41–1.00)	0.046	0.52 (0.24–1.03)	0.077	1.00 (0.50–1.86)	0.990	0.85 (0.32–2.38)	0.742	0.37 (0.32–2.38)	0.150
*Grewia tenax*	0.69 (0.44–1.11)	0.119	0.67 (0.32–1.31)	0.262	1.13 (0.56–2.13)	0.719	1.11 (0.43–3.21)	0.832	0.53 (0.43–3.21)	0.333
*Gisekia africana*	0.37 (0.18–0.78)	0.008	0.25 (0.04–0.88)	0.065	0.18 (0.01–0.86)	0.092	1.44 (0.42–6.44)	0.590	0.00 (0.42–6.44)	1.000
Other	0.89 (0.44–1.86)	0.736	1.16 (0.43–2.88)	0.753	0.70 (0.16–2.17)	0.584	0.64 (0.11–4.26)	0.615	1.02 (0.11–4.26)	0.978
Captures from vegetation by season										
Rainy season	1		1		1		1		1	
Dry season	1.27 (1.05–1.53)	0.012	1.25 (0.95–1.63)	0.106	1.10 (0.82–1.47)	0.506	1.05 (0.67–1.64)	0.844	2.17 (0.67–1.64)	< 0.0001

*RR* Risk ratio

**Table 2:** Plant resting preference by *Culex* spp.: negative binomial regression model results

Variables	Mosquito species							
	*Cx. pipiens*		*Cx. univittatus*		*Cx. poicilipes*		*Cx. ethiopicus*	
	RR (95 % CI)	*P*	RR (95 % CI)	*P*	RR (95 % CI)	*P*	RR (95 % CI)	*P*
Plant species								
*D. kilimandscharicum*	1		1		1		1	
*Commelina forskali*	1.02 (0.51–2.11)	0.947	1.09 (0.51–2.11)	0.738	0.65 (0.29–1.47)	0.292	1.03 (0.29–1.47)	0.935
*Salsola kali*	0.71 (0.31–1.60)	0.400	0.70 (0.31–1.60)	0.233	0.52 (0.21–1.28)	0.149	1.11 (0.21–1.28)	0.795
*Salvadora persica*	0.18 (0.05–0.54)	0.004	0.79 (0.05–0.54)	0.428	0.18 (0.05–0.57)	0.006	1.05 (0.05–0.57)	0.899
*Cadaba ruspolii*	0.43 (0.15–1.13)	0.088	1.13 (0.15–1.13)	0.680	0.31 (0.10–0.93)	0.039	0.62 (0.10–0.93)	0.331
*Mollugo nodicaulis*	0.63 (0.20–1.99)	0.423	1.18 (0.20–1.99)	0.645	0.63 (0.19–2.14)	0.448	1.59 (0.19–2.14)	0.339
*Cyperus giolii*	0.59 (0.16–2.06)	0.398	0.72 (0.16–2.06)	0.449	0.45 (0.11–1.75)	0.245	0.18 (0.11–1.75)	0.112
*Grewia tenax*	0.37 (0.07–1.53)	0.190	0.73 (0.07–1.53)	0.490	0.26 (0.04–1.33)	0.123	0.00 (0.04–1.33)	1.000
*Gisekia africana*	0.28 (0.01–2.40)	0.281	0.00 (0.01–2.40)	0.999	0.27 (0.01–2.61)	0.283	0.00 (0.01–2.61)	1.000
Other	1.11 (0.18–8.62)	0.910	0.51 (0.18–8.62)	0.418	1.08 (0.16–9.94)	0.935	1.16 (0.16–9.94)	0.874
Captures from vegetation by season								
Rainy season	1		1		1		1	
Dry season	1.46 (0.87–2.49)	0.152	1.24 (0.87–2.49)	0.229	1.37 (0.77–2.49)	0.268	1.30 (0.77–2.49)	0.310

*RR* Risk ratio

Mosquitoes collected from vegetation were mainly from the genera *Aedes* and *Culex.* Among the *Aedes* mosquitoes, *Ae. mcintoshi* was the most abundant species captured from all vegetation types followed by *Ae. ochraceus*. The two species were also the most abundant in *D. kilimandscharicum* with average catches of 2.17 and 1.47 per plant for *Ae. mcintoshi* and *Ae. ochraceus,* respectively. This study also assessed the preference for plant species by different mosquito species relative to *D. kilimandscharicum* (Tables 1 and 2). There was a significant difference in captures of *Ae. mcintoshi* from *S.kali*, and *C. giolii* relative to *D. kilimandscharicum* showing high preference of the latter plant species by *Ae. mcintoshi.* Similarly, captures of both *Ae. tricholabis* and *Ae. sudanensis* from *S. kali and C. giolii* relative to *D. kilimandscharicum* were significantly different; whereas *Cx. pipiens* preferred *D. kilimandscharicum* to *S. persica.*

### Parity of vectors of RVF

Our results showed that 1124 (79 %, 95 % CI = 76.8–81.1 %) of mosquitoes were parous. The proportion of parous *Ae. ochraceus* was 80.60 % while the proportion of parous *Ae. mcintoshi* was 77.95 %. Quasibinomial model results indicated that this difference in the numbers of parous mosquitoes between *Ae. ochraceus* and *Ae. mcintoshi* was not significant (OR = 1.04, 95 % CI = 0.71–1.51, *P* = 0.847). The number of parous mosquitoes was significantly greater among those caught during the rainy season than those caught in the dry season (OR = 0.08, 95 % CI = 0.04–0.14, *P* < 0.001). However, the survival rate of the two species did not show any significant difference (OR = 1.05, 95 % CI = 0.71–1.61, *P* = 0.886). There was no impact of collection method on the number of parous mosquitoes (OR = 1.16, 95 % CI = 0.72–1.82, *P* = 0.752). Based on the proportion of parous mosquitoes for each species, we estimated the daily survival rate for each mosquito separately. Daily survival rate *p* was estimated to be 0.93 for *Ae. ochraceus* while the daily survival rate for *Ae. mcintoshi*, was estimated to be 0.92. Our results also showed that difference in survival rate was not significant between the two mosquito species *F*_(1,31)_ = 0.240, *P* = 0.627.

## Discussion

The ability of mosquitoes to withstand environmental stresses and find suitable refuges are adaptations that may contribute to the disease transmission efficiency of these vectors. This study shows that mosquitoes trapped were resting among plants sampled. In addition survival rate was also high among the primary vectors *Ae. mcintoshi* and *Ae. ochraceus*. Plant resting preference and survival of these mosquitoes may potentially influence disease transmission.

Mosquitoes were collected from different plant species, showing that these mosquitoes utilise plants for outdoor resting in the study area. This is supported by the observation in other studies that mosquitoes usually seek shelter in different types of habitats including vegetation [30, 31]. Our study focused on individual plant species with the view of determining their specific roles in mosquito resting behaviour. Generally, the plant species from which the highest numbers of mosquitoes were trapped were *D. kilimandscharicum, C. forskali* and *M. nodicaulis* when compared with other plant species in this study. This suggests the potential for preference for these plant species over others due to the resources that they provide. Mosquitoes could utilise these vegetation to seek protection from extreme temperatures, desiccation and predation in order to enhance their survival [31]. For example, *D. kilimandscharicum* grows in clusters, potentially creating a suitable microclimate beneath the plant that may be preferred by mosquitoes for resting in an otherwise semi-arid environment. These plants may also produce attractants that mosquitoes use to choose resting sites, as has been demonstrated for malaria vectors [32, 33]. Some researchers have also found that mosquitoes often use plants as sources of sugar [34–36], which may also account for observed differences in resting preference.

It is not currently clear why mosquitoes were found in low numbers in some plants, such as *Gisekia africana*. However, some plants are known to produce chemicals that repel insects [37, 38]. The attractive and repellant properties of plants with regard to primary and secondary RVF vectors could provide information useful for a targeted environmental management approach such as the “push-pull” system that has been used successfully for the control of agricultural pests in Africa [39]. Further research to determine the basis for resting site preference is required before such a control tactic could become a reality.

The overall high capture of mosquitoes from vegetation during the dry season may also have been due to reduced vegetation cover in the dry season, consequently making the average capture per plant higher in the dry season than in the rainy season. This observation may also apply to *Ae. sudanensis*, which was caught in significantly higher numbers from plants during the dry season. Other than high plant density, alternative resting sites may have been present in the rainy season resulting in a more even distribution of adults and low average captures per plant in comparison to the dry season.

Overall, most of the mosquitoes trapped were parous, which suggests that the majority of these mosquitoes were able to obtain a blood meal and complete at least one or more egg laying cycles. Parity among mosquitoes may have a bearing on disease circulation. This is because mosquitoes that have already obtained one or more blood meals may have already acquired pathogens from these feeding events, which increases the likelihood that they will transmit these arboviruses when they seek a fresh blood meal. This is consistent with other studies, which reported that mosquito feeding behaviour under different ecological conditions may contribute to its longevity, and consequently influence the transmission of mosquito-borne diseases [40]. Based on our results, *Ae. mcintoshi* and *Ae. ochraceus* are equally capable of identifying suitable hosts, and surviving long enough to develop and lay eggs, then potentially seek another blood meal. This may determine their competence for transmission of arboviruses circulating in north-eastern Kenya. This corroborates the findings of studies conducted elsewhere which documented that vector survival increases their disease transmission potential [40]. This may be further compounded by the fact that a high abundance of these flood water *Aedes* has been reported in north-eastern region of Kenya [41, 42]. The difference in the abundance of the vectors across ecological zones also increases the risk of exposure to infectious bite by these mosquitoes in the region. *Aedes ochraceus* was recently incriminated in the 2006-2007 RVF outbreak in Kenya [17]. Our study suggests that both *Ae. ochraceus* and *Ae. mcintoshi* may have the potential to efficiently acquire several blood meals and potentially initiate circulation of arboviruses including RVFv. Increased geographic expansion of *Ae. ochraceus* despite recent introduction as reported in recent studies [43], may enhance the potential of this vector in circulating arboviruses into diverse ecological zones in East Africa.

More mosquitoes were parous during the rainy season than dry season. This may have been due to suitable climatic conditions such as rainfall and temperature [20, 44–46], as well as higher relative humidity [47, 48], which largely influence mosquito biology. This is consistent with other studies conducted on the ecology of the primary vectors of RVF in this region [17, 41, 42]. Presence of flooded environment and breeding habitats in the study area may have also contributed to the observed high parity in the rainy season than dry season due to the fact that flooded habitats are key larval habitats for these *Aedes* mosquitoes [49]. Low parity rate was observed in the dry season, which may have been due to the extreme dry weather conditions resulting into resorption of follicles by mosquitoes. This may have led to mosquitoes diapausing and retaining eggs or entering dormancy as a way of avoiding dry conditions [50]. In view of our findings, it may be important to incorporate other means of assessing mosquito survival in future studies other than parity in order to make conclusive comparisons of survival between dry and wet seasons. This may form a basis for informed and appropriate mosquito control approach across seasons.

This study reports high survival rate among the two mosquito species *Ae. mcintoshi* and *Ae. ochraceus*. Although the daily survival rate did not significantly differ between the two species, the estimated survival rate could lead to increased opportunity for the incubation of pathogens with long extrinsic incubation periods (EIPs) relative to mosquito lifespan as reported in other studies [51, 52]. High survival rate of these mosquitoes could also increase their vectorial capacity given that they may encounter a number of susceptible hosts in their lifespan when they seek a blood meal. The estimated survival rate (or predicted lifespan) of these primary vectors of RVF in this study could also be sufficiently high to allow incubation arboviruses such as RVF which have been reported to have an extrinsic incubation of up to 14 days [53]. This may enhance their chances of increasing transmission and circulation of arboviruses.

## Conclusion

Survival rate of each of the two primary vectors of RVF reported in this study may provide an indication that these mosquitoes can potentially play important roles in the circulation of diseases in northern Kenya. Differences in the parity of primary vectors of RVF observed in this study between the seasons suggest that these mosquitoes can potentially play important roles in the circulation of diseases in the rainy season. Both primary and secondary vectors of RVF showed resting preferences for certain plant species. Thus, areas dominated with such vegetation may become high-risk zones during epidemics given that the vegetation may provide resources required by the vectors and enhance their survival. The finding that RVF vectors utilise certain plant species for refuges, will in the future help to guide control operations targeting adult mosquitoes during outbreaks to interrupt transmission and minimise virus activity in northern Kenya.

## Abbreviations

CDC, center for disease control; EIP, extrinsic incubation period; LTs, light traps; PBS, phosphate buffered saline; RR, risk ratios; RVF, Rift Valley Fever; RVFv, Rift Valley fever virus.
